# Pharmacogenomics in Diabetes: Population-Specific Insights from Colombia

**DOI:** 10.3390/jpm15100481

**Published:** 2025-10-09

**Authors:** David A. Hernandez-Paez, Johana Galván-Barrios, Kevin Fernando Montoya-Quintero, Indiana Luz Rojas Torres

**Affiliations:** 1 Center for Meta-Research and Scientometrics in Biomedical Sciences, Barranquilla 080002, Colombia; davidtdep312@gmail.com; 2 Grupo Prometheus y Biomedicina Aplicada a las Ciencias Clinicas, School of Medicine, Universidad de Cartagena, Cartagena 130001, Colombia; 3 Biomedical Scientometrics and Evidence-Based Research Unit, Department of Health Sciences, Universidad de la Costa, Barranquilla 080002, Colombia; 4 Facultad de Ciencias para la Salud, Universidad de Manizales, Manizales 170001, Colombia; kmontoya@umanizales.edu.co; 5 Centro de Investigaciones en Ciencias de la Vida, Facultad de Ciencias de la Salud, Universidad Simón Bolívar, Barranquilla 080001, Colombia

**Keywords:** pharmacogenetics, genomics, diabetes mellitus, glucose metabolism disorders, cardiovascular diseases

## Abstract

**Background:** Pharmacogenomics offers critical insights into interindividual variability in drug response, especially in complex diseases such as diabetes mellitus. However, most pharmacogenomic evidence is derived from populations of European ancestry, limiting its applicability in admixed and underrepresented populations. In Colombia, the lack of population-specific data hampers the implementation of precision medicine strategies in diabetes care. The aim of this study was to identify pharmacogenomic variants significantly associated with diabetes and exhibiting differential allele frequencies between Colombian populations of African and European ancestry. **Methods**: We extracted 115 variant annotations related to diabetes from PharmGKB and filtered them for statistical significance and availability of allele frequency data. Fourteen single-nucleotide polymorphisms (SNPs) were compared across five Colombian populations using the CÓDIGO genomic diversity database. Principal component analysis (PCA) was performed to assess genetic clustering, and Pearson correlation coefficients were used to assess pharmacogenomic similarity. **Results**: PCA revealed distinct genetic clustering patterns that aligned with geographical distribution and ancestral origins. Pharmacogenomic divergence was observed between African and European ancestry groups in Colombia, with certain SNPs (e.g., rs8192675-C for metformin, rs7754840-C for DPP-4 inhibitors) showing 2- to 3-fold higher frequency in African ancestry populations. The bibliometric analysis revealed that 76.1% of studies originated from high-income countries and 68.4% of participants were of European ancestry. No studies originated from Africa or low-income countries. **Conclusions**: Marked ancestry-based differences in pharmacogenomic variant frequencies in Colombian populations may impact drug efficacy and risk of diabetes. The global literature shows a strong geographic and economic bias, underscoring the need for inclusive, population-specific pharmacogenomic research. These findings offer a foundation for implementing precision diabetes therapies in Latin America and advancing equitable genomic medicine.

## 1. Introduction

Diabetes mellitus (DM) is one of the most prevalent chronic diseases worldwide, affecting over 530 million people and imposing an increasingly significant burden on healthcare systems, particularly in low- and middle-income countries (LMICs) such as Colombia [1]. Its chronic and multifactorial nature contributes to considerable morbidity, disability, premature mortality, and escalating costs associated with long-term pharmacological treatment and complications [2]. Despite advancements in therapeutic strategies, a substantial proportion of individuals with diabetes fail to achieve optimal glycemic control, which underscores the interindividual variability in drug response [3]—a phenomenon increasingly explained by pharmacogenomic factors [4].

Pharmacogenomics—the study of how genetic variations affect drug metabolism, efficacy, and toxicity—represents a critical pillar of precision medicine. This emerging field offers a pathway toward individualizing treatment by integrating genomic data to predict therapeutic response, minimize adverse effects, and optimize clinical outcomes [4]. In diabetes care, pharmacogenomics has shown promise in tailoring treatments involving commonly prescribed agents such as metformin, sulfonylureas, thiazolidinediones, and dipeptidyl peptidase-4 (DPP-4) inhibitors, among others [5]. Several genetic variants, particularly single-nucleotide polymorphisms (SNPs) in genes such as TCF7L2, SLC2A2, and CYP3A5, have been linked to the altered pharmacokinetics and pharmacodynamics of these medications, offering new insights into mechanisms underlying therapeutic variability [6].

Although pharmacogenomic research in diabetes has expanded globally, much of the existing evidence stems from populations of European or Asian ancestry [7]. This disproportionate representation introduces significant limitations when extrapolating findings to admixed or underrepresented populations, particularly those in Latin America [8]. Genetic diversity in populations such as those in Colombia, where complex admixture patterns involving Indigenous, European, and African ancestries exist [9], may influence the allele frequencies of pharmacogenetically relevant variants and, consequently, drug response [10]. However, little is known about the distribution of these variants in Latin American populations or how this genetic diversity may affect pharmacological management of diabetes in clinical settings.

In Colombia, and more broadly in South America, the field of pharmacogenomics remains underdeveloped and poorly integrated into clinical practice [11]. Factors contributing to this knowledge gap include limited access to genomic infrastructure, the underrepresentation of local populations in global databases, and a lack of context-specific studies examining pharmacogenomic variants in relation to the unique ancestry profiles of these regions [12,13]. Consequently, the application of pharmacogenomics in clinical care continues to be largely aspirational rather than operational in LMICs. This disparity not only impedes the translation of research into practice but also exacerbates health inequities in the management of chronic diseases such as diabetes.

Addressing this gap requires the generation of local evidence that characterizes the genetic architecture of pharmacogenomic markers in diverse populations [14]. Colombia, with its rich genetic heterogeneity and well-characterized biogeographical subgroups, offers a unique opportunity to explore how ancestry modulates pharmacogenomic profiles in diabetes [9]. Understanding the differences in allele frequencies of pharmacogenetically relevant SNPs across ancestral groups is essential for advancing precision medicine in endocrinology and designing equitable therapeutic interventions.

This study aimed to (1) identify pharmacogenomic variants related to diabetes that show significant differences in allele frequencies between Colombian populations of African and European ancestry, and (2) evaluate the geographic, economic, and bibliometric distribution of published studies on diabetes pharmacogenomics. In addressing these aims, we seek to uncover overlooked disparities in the global pharmacogenomic literature and highlight the need for a greater representation of underexplored populations in precision medicine research.

## 2. Materials and Methods

### 2.1. Study Design

This study utilized a cross-sectional design.

### 2.2. Data Collection

A search of the PharmGKB database [15] for “Diabetes mellitus” (PharmGKB ID: PA443886), conducted on 26 February 2025, yielded 115 variant annotations from 42 studies (Supplementary Material S1). These annotations included information on genes, alleles/genotypes, significance of associations, biogeographical groups, the number of cases and controls included in each study, related drugs, phenotypes (e.g., increased or decreased toxicity, efficacy, metabolism/pharmacokinetics, among others), and the PMID/PMCID of the articles.

From this initial set of annotations, we selected those meeting three criteria: (1) an association with a single SNP allele, (2) statistical significance (*p* < 0.05), and (3) a reported allele frequency. This filtering process explicitly excluded records corresponding to genotypes (e.g., rs2368564-CT) or gene variants (e.g., CYP3A5*1). We consulted the Consortium for Genomic Diversity, Ancestry, and Health in Colombia (CÓDIGO) database [16] to obtain allele frequencies for the identified SNPs in two populations with predominantly African ancestry, Palenque (PLQ, n = 34, 84% African ancestry) and Chocó (CHG, n = 96, 76%), and three populations with predominantly European ancestry from Antioquia, ATQCES (n = 404, 50.5% European ancestry), ATQPGC (n = 624, 55% European ancestry), and CLM (n = 96, 62.9% European ancestry). These five populations were selected based on their predominant ancestry proportion (>50%) and comprehensive data availability in the database, as other populations lacked sufficient reported allele frequencies.

The CÓDIGO database is a national initiative that integrates genomic, ancestry, and health data from diverse Colombian populations, aiming to characterize the country’s genetic diversity and support biomedical, epidemiological, and public health research. Its scope encompasses multiple population groups with distinct ancestral backgrounds, and it has evolved through the collection and harmonization of whole-genome, whole-exome, and genotyping data, enabling population-specific allele frequency estimation.

Allele frequencies for the PLQ and ATQPGC populations were estimated using whole-genome sequencing, while those for the remaining populations were obtained using either whole-exome sequencing or whole-genome genotyping. The methods used for managing and calculating the allele frequencies from the raw sequencing data are described in detail by Mariño-Ramírez et al. [16]. The CÓDIGO database provides summary data rather than raw genomic, exomic, or genotype data. For SNPs with known risk alleles based on PharmGKB variant annotation, we extracted specific allele frequencies for each population. Details regarding the allele counts are available in the CÓDIGO database [17]. This refined dataset included a total of 14 variant annotations (Supplementary Material S2), which were subsequently used for all further analyses.

### 2.3. Statistical Analysis

Using the refined dataset comprising 14 variant annotations, we conducted Pearson’s correlation analyses between the reported allele frequencies across populations. For each pairwise comparison, variants with at least one missing value were excluded. To further elucidate potential population stratification patterns in pharmacogenomic profiles, we performed principal component analysis (PCA) on the allele frequency data across the five ancestry populations. The PCA was implemented using a probabilistic approach (PPCA) to accommodate missing values (11.4% of the dataset), allowing for the retention of all 14 pharmacogenetically significant variants in the analysis. The data matrix was transposed to position populations as observations and variants as variables, enabling the identification of clustering patterns among populations based on their pharmacogenomic allele frequency profiles. Principal components were derived without scaling but with mean centering, preserving the relative magnitude of frequency differences among variants. We extracted the first two principal components, which collectively accounted for 97.9% of the total variance (PC1: 84.0%, PC2: 13.8%).

Then, we computed the mean allele frequency for each ancestry group, namely African ancestry (PLQ and CHG) and European ancestry (ATQCES, ATQPGC, and CLM). Finally, we aggregated the total number of cases and controls according to the biogeographical group reported in each of the studies, classifying them as European, African, American, Asian, Mixed, or Unknown (Supplementary Table S1).

### 2.4. Bibliometric Analysis

Metadata for each of the 42 studies corresponding to the identified variant annotations was retrieved using the PMID/PMCID through the NCBI Entrez API, Unpaywall API, Crossref API, and the SCImago Journal Rank database. This process provided information on the document title, publication date, first author and their country of affiliation, access type (open or closed), number of citations, H-index, and journal quartile. The country of affiliation of the first author was used to infer the corresponding World Health Organization (WHO) region [18] and World Bank income group [19], which served as two key dimensions in the descriptive bibliometric analysis.

All the analyses were conducted in R (v4.4.0) [20]. The script and datasets, along with detailed annotations, are available at https://doi.org/10.5281/zenodo.16413780.

## 3. Results

### 3.1. Bibliometric Landscape

**Figure 1:**
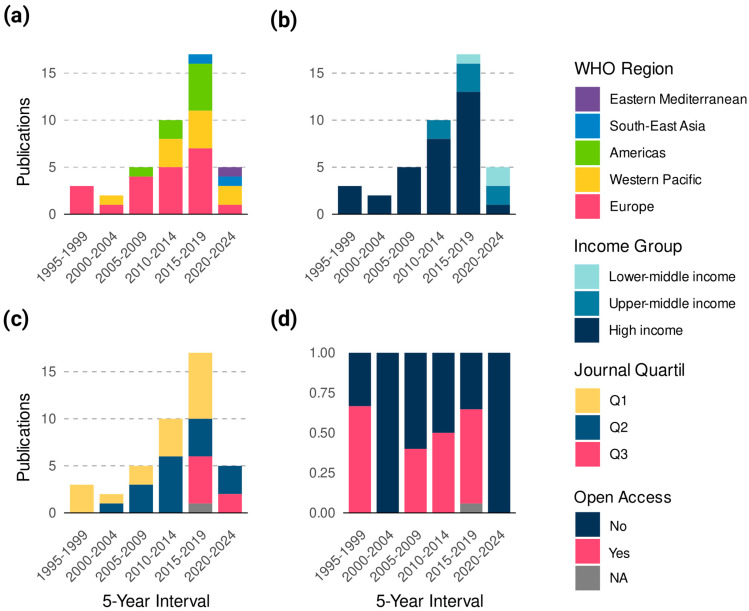
Temporal trends and characteristics of pharmacogenomics studies in breast cancer indexed in PharmGKB (n = 42). (**a**) Distribution of publications by WHO region across 5-year intervals. (**b**) Distribution of studies by World Bank income group. (**c**) Distribution of publications by journal quartile (Q1–Q3). (**d**) Proportion of studies published as open access over time.

All 115 variants identified in the first step of the search methodology were reported across 42 studies, representing all studies on pharmacogenomics of diabetes registered in PharmGKB. The earliest of these publications was from 1996 in BMJ by Parving et al. [21]; however, its results were not included in the main analysis due to not meeting the inclusion criteria. Among all studies, 50% originated from the European region (Figure 1a and Table 1) and 76.1% from high-income countries (Figure 1b and Table 2). These two categories also accounted for the majority of citations, with Europe contributing 1046 citations (49.8%) and high-income countries contributing 1322 citations (41.3%). Both categories showed the lowest open access ratios among groups with more than three publications, with values of 0.75 for Europe and 1.13 for high-income countries. Regarding journal ranking, 41.4% of the articles were published in Q1 journals and another 41.4% were published in Q2 journals (Figure 1c). Overall, 52.3% of the articles were not open access (Figure 1d). Interestingly, no publications came from Africa or low-income countries. The average H-index of journals was 203.7 (SD: 159.3) for the European region and 196.1 (SD: 144.4) for high-income countries. The three journals with the highest number of publications were *The Pharmacogenomics Journal* (n = 7), *Pharmacogenetics and Genomics* (n = 5), and *The European Journal of Clinical Pharmacology* (n = 4) (Supplementary Material S1). Among the 42 studies, only the results from 14 met the inclusion criteria for the main analysis (Supplementary Material S2).

### 3.2. The Role of Genetic Ancestry in Colombia

**Figure 2:**
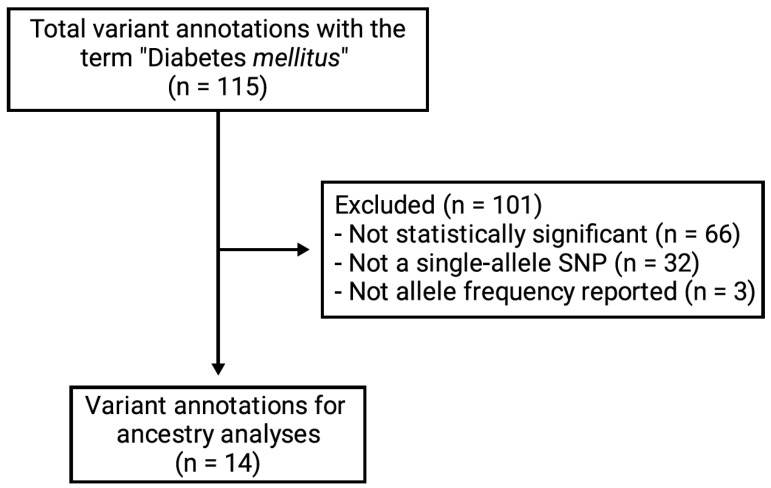
Flowchart illustrating the inclusion methodology for the variant annotations.

**Figure 3:**
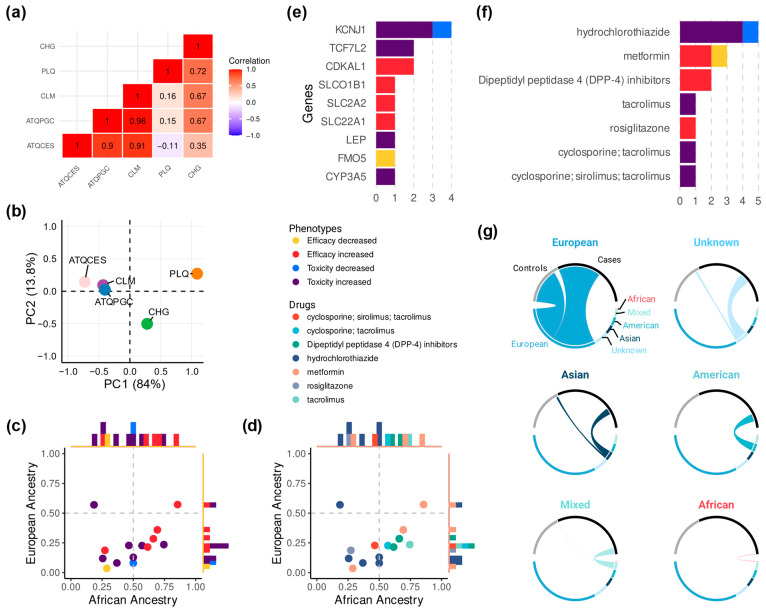
Current knowledge and research gaps in Colombian diabetes pharmacogenomics. (**a**) Correlation matrix showing Pearson’s coefficients of the pharmacogenomic profiles of five populations based on allele frequencies. (**b**) Principal component analysis of allele frequency profiles across five Colombian populations. (**c**) Scatter plot of significant SNPs, colored by associated phenotype. (**d**) Scatter plot of significant SNPs, colored by related drug. (**e**) Top seven drugs and (**f**) top nine genes with the highest number of significant SNPs, colored by phenotype. (**g**) Chord plots representing the proportion of cases (n = 37,066) and controls (n = 19,830) by ancestry groups reported across all identified studies (n = 42).

We ultimately extracted 14 variant annotations that met the inclusion criteria (Figure 2), which were used in the subsequent analyses. Pearson’s correlation coefficients were very similar among Colombian populations with predominant European ancestry (i.e., ATQCES, ATQPGC, and CLM), each showing values greater than or equal to 0.9, indicating a highly similar pharmacogenomic profile in terms of allele frequency (Figure 3a). A similar profile was observed between the Colombian populations with predominant African ancestry, with a Pearson coefficient of 0.72 between PLQ and CHG. In contrast, comparisons between PLQ and the three European-predominant populations showed correlations near zero, indicating low similarity in pharmacogenomic profiles. CHG showed moderate correlations with CLM (r^2^ 0.67) and ATQPGC (r^2^ 0.67), and a lower correlation with ATQCES (r^2^ 0.35).

Principal component analysis further substantiated these population stratification patterns (Figure 3b). The first two principal components collectively accounted for 97.85% of the total variance in allele frequencies across populations (PC1: 84.02%, PC2: 13.83%), indicating robust discrimination of pharmacogenomic profiles. Consistent with the correlation findings, ATQCES, ATQPGC, and CLM formed a distinct cluster in the PCA space, all displaying negative values on PC1 (ATQCES: −0.73; CLM: −0.43; ATQPGC: −0.40), confirming their pharmacogenomic similarity. Conversely, PLQ exhibited the most divergent profile with a strongly positive PC1 score (1.09), while CHG occupied an intermediate position with a moderately positive PC1 value (0.28) but strongly negative PC2 value (−0.50).

We then calculated the average allele frequencies for the three predominantly European populations and the two predominantly African populations. Several SNPs were found to be more frequent in the African ancestry group, while only one SNP showed higher frequency in the European ancestry group (Figure 3c,d). Among the SNPs predominant in African ancestry, rs7754840-C, associated with increased response to dipeptidyl peptidase 4 (DPP-4) inhibitors in people with diabetes mellitus, was present in 66.1% of the mean African allele frequency and 28.4% of the mean European allele frequency. Similarly, rs7756992-G, associated with the same drug and phenotype, had a mean frequency of 61.5% in the African group and 21.4% in the European group. Another predominantly African SNP was rs8192675-C, associated with increased response to metformin in people with diabetes mellitus, with a mean frequency of 69.3% in the African group and 35.9% in the European group (Table 3).

Some of the identified drugs were not directly used to treat diabetes but had associated SNPs linked to an increased risk of developing diabetes when administered to individuals carrying specific alleles. Among these, the SNP rs7917983-T was associated with an increased likelihood of new-onset diabetes when treated with hydrochlorothiazide in individuals with hypertension. This allele was present in 18.5% of the African group and 57% of the European group. Similarly, the SNP rs776746-T was associated with an increased likelihood of post-transplant diabetes in patients with kidney transplantation treated with tacrolimus. This allele was frequent in the African group (74.5%) and less prevalent in the European group (23.5%) (Table 3).

### 3.3. A Summary of Drugs and Genes with the Most Reported Associations

In pharmacogenomics related to diabetes, the drug with the highest number of significant genetic associations was hydrochlorothiazide, predominantly linked to an increased likelihood of new-onset diabetes in individuals with hypertension. Metformin and DPP-4 inhibitors ranked second and third, with most associations linked to increased response to them in people with diabetes mellitus (Figure 3e,f). Regarding genes, the top three with the highest number of significant associations were KCNJ1, TCF7L2, and CDKAL1, primarily associated with phenotypes of an increased risk of new-onset diabetes and efficacy (Figure 3e,f).

### 3.4. What We Overlook: The European Bias

A total of 56,896 individuals were included as either cases (n = 37,066; 65.1%) or controls (n = 19,830; 34.8%) across the 42 studies retrieved through our search strategy on PharmGKB. Of these, 38,917 (68.4%) were of European ancestry, representing the majority. Individuals of Asian and American ancestry accounted for 4216 (7.4%) and 3865 (6.7%), respectively. A total of 131 individuals of African ancestry (0.3%) were reported, all of whom were classified as cases, with no controls identified. The remaining participants either had no reported ancestry (n = 6474; 11.3%) or were classified under mixed ancestry groups (n = 3293) (Figure 3g and Supplementary Material S1).

## 4. Discussion

The bibliometric analysis of the 42 publications on diabetes pharmacogenomics revealed a clear concentration of scientific output in high-income countries, particularly in the European region, which accounted for over 50% of studies. More than three-quarters of the included studies originated from high-income nations, underscoring a systemic imbalance in the global production and dissemination of pharmacogenomic knowledge. The underrepresentation of LMICs, especially in Africa and Latin America, not only limits our understanding of genetic variation in these populations but also compromises the global applicability of pharmacogenomic findings [26].

Although a significant portion of the articles were published in high-impact journals, over half were not open access, further compounding barriers to knowledge dissemination in resource-constrained settings [27]. This geographic and economic skew in knowledge production directly impacts the clinical implementation of pharmacogenomics in Colombia in three crucial ways. First, the predominant European ancestry focus (68.4% of study participants) means that the pharmacogenomic variants we found to be significantly more prevalent in Colombian African ancestry populations (such as rs7754840-C for DPP-4 inhibitors and rs8192675-C for metformin) remain understudied in clinical outcomes research. Second, the limited representation of Latin American researchers in this field (as our bibliometric analysis revealed) correlates with the minimal investigation of the specific admixture patterns in Colombian populations that our genetic analysis identified as pharmacogenomically relevant. Third, the lack of open access literature creates a direct implementation barrier for Colombian clinicians seeking to apply pharmacogenomic principles in diabetes care, particularly for the ancestry-specific variants we identified. This dual bias, both economic and geographic, reinforces inequities in the generation and application of pharmacogenomic evidence and hinders the implementation of precision medicine strategies where they are most needed [13,28].

A key contribution of this study lies in the identification of ancestry-related pharmacogenomic disparities among Colombian populations. The high Pearson correlation coefficients (>0.9) observed between the three populations of predominantly European ancestry suggest a shared pharmacogenomic profile. In contrast, low or moderate correlations between these populations and those with predominant African ancestry (Palenque and Chocó) highlight significant genetic divergence.

This finding carries substantial clinical implications [29]. Specific SNPs associated with increased drug efficacy, such as rs7754840-C and rs8192675-C for DPP-4 inhibitors and metformin, respectively, were notably more frequent in African ancestry groups. This may partly explain interindividual variability in therapeutic responses, even when standard treatment protocols are followed. Conversely, SNPs linked to higher diabetes risk under certain pharmacological treatments, such as rs776746-T (tacrolimus) and rs7917983-T (hydrochlorothiazide), also displayed ancestry-specific patterns.

Such insights underscore the necessity of integrating ancestry-informed pharmacogenomic data into clinical decision-making [30]. Failing to do so may perpetuate suboptimal care in genetically diverse populations and may lead to adverse drug events or therapeutic inefficacy, particularly in contexts of comorbidity such as hypertension or kidney transplantation [31,32].

The overrepresentation of European ancestry participants (68.4%) in the analyzed studies reflects a widespread limitation in pharmacogenomic research. Only 131 individuals of African ancestry were reported, exclusively as cases, with no control data available, raising concerns about the methodological robustness and generalizability of findings. This imbalance restricts the external validity of research outcomes and potentially propagates inaccurate assumptions when applied to underrepresented populations [22].

By focusing predominantly on homogeneous genetic backgrounds, global pharmacogenomic research may fail to capture the true extent of interethnic variation in drug metabolism and response. Such oversight risks widening rather than narrowing existing health disparities. As pharmacogenomics becomes increasingly embedded in therapeutic algorithms, the lack of representative data from admixed and minority populations must be viewed as a major challenge to the equity and effectiveness of precision medicine.

### 4.1. Gene–Variant Context and Mechanistic Links to Phenotypes

The variants summarized in Table 3 fall into genes with established roles in glucose homeostasis, β-cell physiology, or drug metabolism. CDKAL1 encodes a tRNA methylthiotransferase-like enzyme implicated in β-cell function. Risk alleles at rs7754840-C and rs7756992-G have been associated with impaired first-phase insulin secretion and reduced β-cell KATP-channel responsiveness to glucose; these alleles predicted greater HbA1c reduction after the initiation of a DPP-4 inhibitor, consistent with incretin-mediated rescue of β-cell secretory defects [23].

SLC2A2 encodes GLUT2, a key hepatocellular glucose transporter. In a three-stage genome-wide association study (GWAS) meta-analysis, the rs8192675-C allele was associated with a greater metformin-induced reduction in HbA1c and is the top cis-eQTL for SLC2A2 in the liver, corresponding to lower expression. This supports a hepatic mechanism in which metformin preferentially benefits carriers with a GLUT2-related glycemic defect [24].

TCF7L2 encodes a WNT-pathway transcription factor that regulates proglucagon transcription in L-cells and modulates incretin signaling and islet function. In the INVEST-GENES study, the rs7917983 variant showed the strongest hydrochlorothiazide (HCTZ) × genotype interaction, conferring an increased risk of new-onset diabetes mellitus among HCTZ-treated hypertensive patients. This finding aligns with a biologically plausible link between TCF7L2-mediated incretin/islet pathways and thiazide-induced dysglycemia [25].

CYP3A5 encodes a cytochrome P450 enzyme responsible for tacrolimus metabolism; functional variation at rs776746 determines enzyme expression status and systemic drug exposure. In renal transplant recipients receiving tacrolimus, the rs776746 genotype was significantly associated with post-transplant diabetes mellitus, consistent with the calcineurin-inhibitor mechanism whereby higher exposure or genotype-driven dosing differences impair β-cell growth and function via disruption of the calcineurin–NFAT signaling axis [33].

### 4.2. Knowledge Gaps as Research Opportunities

This study highlights several critical gaps in the pharmacogenomics of diabetes. First, there is a striking lack of original studies from Latin America—a region with high diabetes prevalence and substantial genetic diversity. Second, most existing studies do not include longitudinal clinical follow-up or validate associations in diverse, real-world settings. Third, there is little exploration of the interaction between genetic variants and environmental or sociocultural factors, which may modulate therapeutic efficacy. Additionally, epigenetic modifications, nutrigenomics, microbiomes, protein interactions, exosomes, and metabolomics represent a critical but understudied layer of pharmacogenomic regulation that may significantly impact drug response [34]

The bibliometric patterns we identified represent more than descriptive statistics—they explain why the ancestry-specific pharmacogenomic profiles we found in Colombian populations remain clinically underutilized. The concentration of research in high-income countries directly limits the generation of clinically actionable data for the variants we found to differ significantly between ancestry groups. Thus, addressing the bibliometric disparities we documented would directly enhance the clinical applicability of the genetic differences we observed between Colombian ancestry groups.

These gaps should not be viewed solely as limitations but as opportunities for future research [35,36,37]. Local genomic initiatives, such as the CÓDIGO project in Colombia, provide an invaluable platform for advancing population-specific pharmacogenomics. Collaborative, cross-regional efforts are also essential to decentralize scientific production and ensure that precision medicine is both scientifically valid and globally relevant.

### 4.3. Contribution to Precision Medicine, Endocrinology, and Diabetes Care

This study represents one of the first efforts to characterize pharmacogenomic profiles of diabetes across ancestrally diverse Colombian populations. By integrating allele frequency data from a national genomic database with meta-research insights into the literature landscape, it offers a dual contribution: highlighting biologically meaningful differences in variant prevalence and exposing systemic biases in the global pharmacogenomic knowledge base.

These findings underscore the imperative to build a more inclusive framework for precision medicine, one that recognizes the heterogeneity of genetic backgrounds and the importance of contextualized, population-specific evidence. In the field of endocrinology, where diabetes is increasingly complex and heterogeneous, incorporating pharmacogenomics into therapeutic planning can improve treatment effectiveness, reduce adverse effects, and support individualized care [23,24,38].

Ultimately, by revealing overlooked disparities and underexplored variants, this study advances the vision of an equitable and representative pharmacogenomics, one that not only refines treatment but also ensures that the promise of precision medicine reaches those who have historically been left behind [25,33].

This study has inherent limitations. First, the analysis was restricted to variant annotations available in the PharmGKB database, which may not fully capture the entire landscape of pharmacogenomic variants associated with diabetes, particularly those identified in more recent or unpublished studies. Second, the allele frequency comparisons were limited to five Colombian populations available in the CÓDIGO database, which, while diverse, may not represent the full genomic variability of the Colombian population or other Latin American groups. Additionally, the study relied on publicly available summary-level data without access to individual-level clinical outcomes, precluding assessments of gene–drug–response interactions in real-world settings. Furthermore, while our findings reveal ancestry-related differences in allele frequencies of pharmacogenetic variants, they do not directly establish clinical response differences in Colombian populations. These allele frequency differences provide a foundation for precision medicine but require clinical validation to confirm their impact on treatment outcomes. Accordingly, these findings are hypothesis-generating rather than causal, and we explicitly call for prospective replication in Colombian cohorts using individual-level genotypes linked to longitudinal clinical outcomes to test these allele-frequency signals against drug response and toxicity. However, these limitations are offset by the study’s strengths, including the integration of high-quality, curated pharmacogenomic and population-specific genomic data, a rigorous selection of variants with demonstrated statistical significance, and a comprehensive bibliometric analysis that contextualizes the global distribution of knowledge. This multidimensional approach provides a robust foundation for future research and highlights underrepresented areas [39,40] where pharmacogenomic evidence is critically needed.

## 5. Conclusions

This study revealed distinct pharmacogenomic profiles between ancestry groups within Colombia, with several variants, particularly those linked to an increased efficacy of metformin and DPP-4 inhibitors, being more prevalent in African ancestry populations. This suggests that ancestry-informed pharmacogenomic data may be critical for optimizing therapeutic decisions and minimizing adverse drug reactions in ethnically diverse settings. Also, we discovered European and high-income country bias in the generation and dissemination of pharmacogenomic evidence, highlighting a major gap in representation for populations from Latin America and other low- and middle-income regions. These disparities underscore the urgent need to promote more inclusive research agendas that account for genetic and contextual diversity.

## Figures and Tables

**Table 1:** General characteristics of articles on diabetes pharmacogenomics by region (N = 42).

	Americas	Europe	Western Pacific	South-East Asia	Eastern Mediterranean	Africa	*p*-Value
Publications (%)	8 (19)	21 (50)	10 (23.8)	2 (0.04)	1 (0.02)	0	
Total Citations (Avg per Paper)	106 (13.2)	1046 (49.8)	303 (30.3)	19 (9.5)	0	0	0.04 ^a^
Avg H-index (SD)	143 (45.1)	203.7 (159.3)	220.9 (147.9)	73.5 (27.5)	99 (0)	0	0.2 ^a^
Open Access/No Open Access (ratio)	6/2 (3)	9/12 (0.75)	3/7 (2.4)	1/1 (1)	0/1 (0)	0	0.18 ^b^
**Journal quartile (%) (n = 41)**							0.14 ^b^
Q1	2 (28.5)	9 (42.8)	6 (60)	0	0	0	
Q2	4 (57.1)	10 (47.6)	2 (20)	0	1 (0)	0	
Q3	1 (14.2)	2 (0.9)	2 (20)	2 (100)	0	0	
Q4	0 (0)	0 (0)	0	0	0	0	

^a^ Kruskal–Wallis H test; ^b^ Fisher’s exact test.

**Table 2:** General characteristics of articles on diabetes pharmacogenomics by income group (N = 42).

	High Income	Upper-Middle Income	Lower-Middle Income	Low-Income	*p*-Value
Publications (%)	32 (76.1)	7 (16.6)	3 (0.71)	0	
Total Citations (Avg per Paper)	1322 (41.3)	133 (19)	19 (6.3)	0	0.03 ^a^
Avg H-index (SD H-index)	196.1 (144.4)	201.2 (140.1)	82 (24.4)	0	0.09 ^a^
Open Access/No Open Access (ratio)	17/15 (1.13)	5/2 (2.5)	1/2 (0.5)	0	1 ^b^
**Journal quartile (%) (n = 41)**					0.04 ^b^
Q1	14 (45.1)	3 (42.8)	0	0	
Q2	14 (45.1)	2 (28.5)	1 (33.3)	0	
Q3	3 (0.96)	2 (28.5)	2 (66.6)	0	
Q4	0	0	0	0	

^a^ Kruskal–Wallis H test; ^b^ Fisher’s exact test.

**Table 3:** Pharmacogenomic variants with differential allele frequencies by ancestry and their associated drug effects.

Gene	SNP	Drug	Mean African AF (%)	Mean European AF (%)	Phenotype	Reference
**Efficacy, increased**						
CDKAL1	rs7754840-C	DPP-4 inhibitors	66.1	28.4	↑ HbA1c reduction	[22]
CDKAL1	rs7756992-G	DPP-4 inhibitors	61.5	21.4	↑ HbA1c reduction	[22]
SLC2A2	rs8192675-C	Metformin	69.3	35.9	↑ HbA1c reduction	[23]
**Toxicity, increased**						
TCF7L2	rs7917983-T	Hydrochlorothiazide	18.5	57	↑ NODM risk	[24]
CYP3A5	rs776746-T	Tacrolimus	74.5	23.5	↑ PTDM risk	[25]

AF: Allele frequency. NODM: New-onset diabetes mellitus. PTDM: Post-transplant diabetes mellitus. ↑: Increased.

## Data Availability

The script and datasets, along with detailed annotations, are available at https://doi.org/10.5281/zenodo.16413780.

## Supplementary Materials

The following supporting information can be downloaded at: https://www.mdpi.com/article/10.3390/jpm15100481/s1, Supplementary Table S1: Biogeographical groups identified. Supplementary Material S1: Total of variant annotations reported across studies. Supplementary Material S2: Dataset included the total of variant annotations.

## Abbreviations

The following abbreviations are used in this manuscript:

ATQCES	European ancestry from Antioquia #1
ATQPGC	European ancestry from Antioquia #2
CHG	Chocó
CLM	Colombians in Medellin
CÓDIGO	Consortium for Genomic Diversity, Ancestry, and Health in Colombia
DM	Diabetes mellitus
DPP-4	Dipeptidyl peptidase-4
LMICs	Low- and middle-income countries
PLQ	Palenque
SNPs	Single-nucleotide polymorphisms
WHO	World Health Organization
