# Supplementary material for: Pharmacogenomics in Diabetes: Population-Specific Insights from Colombia
Source: J Pers Med. 2025 Oct 9;15(10):481. doi: 10.3390/jpm15100481 (PMC12565577; doi:10.3390/jpm15100481)
Supplement: Supplementary file 1 [file jpm-15-00481-s001.zip › Supplementary Table S1.pdf]

## Supplementary Table S1. Biogeographical groups identified.

| Ancestry | Biogeographical group reported | N. of studies |
|---|---|---|
| African | African American/Afro-Caribbean | 1 |
| American | American | 1 |
| | Latino | 9 |
| Asian | Central/South Asian | 2 |
| | East Asian | 24 |
| | Near Eastern | 2 |
| European | European | 38 |
| Mixed | Multiple groups | 4 |
| Unknown | Unkown | 34 |
